# iDNA6mA-Rice: A Computational Tool for Detecting N6-Methyladenine Sites in Rice

**DOI:** 10.3389/fgene.2019.00793

**Published:** 2019-09-10

**Authors:** Hao Lv, Fu-Ying Dao, Zheng-Xing Guan, Dan Zhang, Jiu-Xin Tan, Yong Zhang, Wei Chen, Hao Lin

**Affiliations:** ^1^Key Laboratory for Neuro-Information of Ministry of Education, School of Life Science and Technology, Center for Informational Biology, University of Electronic Science and Technology of China, Chengdu, China; ^2^Innovative Institute of Chinese Medicine and Pharmacy, Chengdu University of Traditional Chinese Medicine, Chengdu, China

**Keywords:** N6-methyladenine, mono-nucleotide binary encoding, random forest, cross-validation, web-server

## Abstract

DNA N6-methyladenine (6mA) is a dominant DNA modification form and involved in many biological functions. The accurate genome-wide identification of 6mA sites may increase understanding of its biological functions. Experimental methods for 6mA detection in eukaryotes genome are laborious and expensive. Therefore, it is necessary to develop computational methods to identify 6mA sites on a genomic scale, especially for plant genomes. Based on this consideration, the study aims to develop a machine learning-based method of predicting 6mA sites in the rice genome. We initially used mono-nucleotide binary encoding to formulate positive and negative samples. Subsequently, the machine learning algorithm named Random Forest was utilized to perform the classification for identifying 6mA sites. Our proposed method could produce an area under the receiver operating characteristic curve of 0.964 with an overall accuracy of 0.917, as indicated by the fivefold cross-validation test. Furthermore, an independent dataset was established to assess the generalization ability of our method. Finally, an area under the receiver operating characteristic curve of 0.981 was obtained, suggesting that the proposed method had good performance of predicting 6mA sites in the rice genome. For the convenience of retrieving 6mA sites, on the basis of the computational method, we built a freely accessible web server named iDNA6mA-Rice at http://lin-group.cn/server/iDNA6mA-Rice.

## Introduction

Methylated bases, such as N4-methylcytosine (4mC), N6-methyladenine (6mA), and 5-methylcytosine (5mC), exist in genomic DNA of diverse species (Cheng, 1995; Ratel et al., 2006). All these DNA methylation modifications play important roles in controlling many biological functions (Tang et al., 2018b). As an epigenetic mechanism, DNA methylation refers to a process that methyl groups are transferred to DNA molecules and is essential in the normal development of organisms (Bergman and Cedar, 2013; Smith and Meissner, 2013; von Meyenn et al., 2016). Through DNA methylation, the activity of a DNA segment can be changed without changing its sequence. For example, gene transcription can be repressed when DNA methylation occurs at its promoter (Bird, 1992).

**Figure 1:**
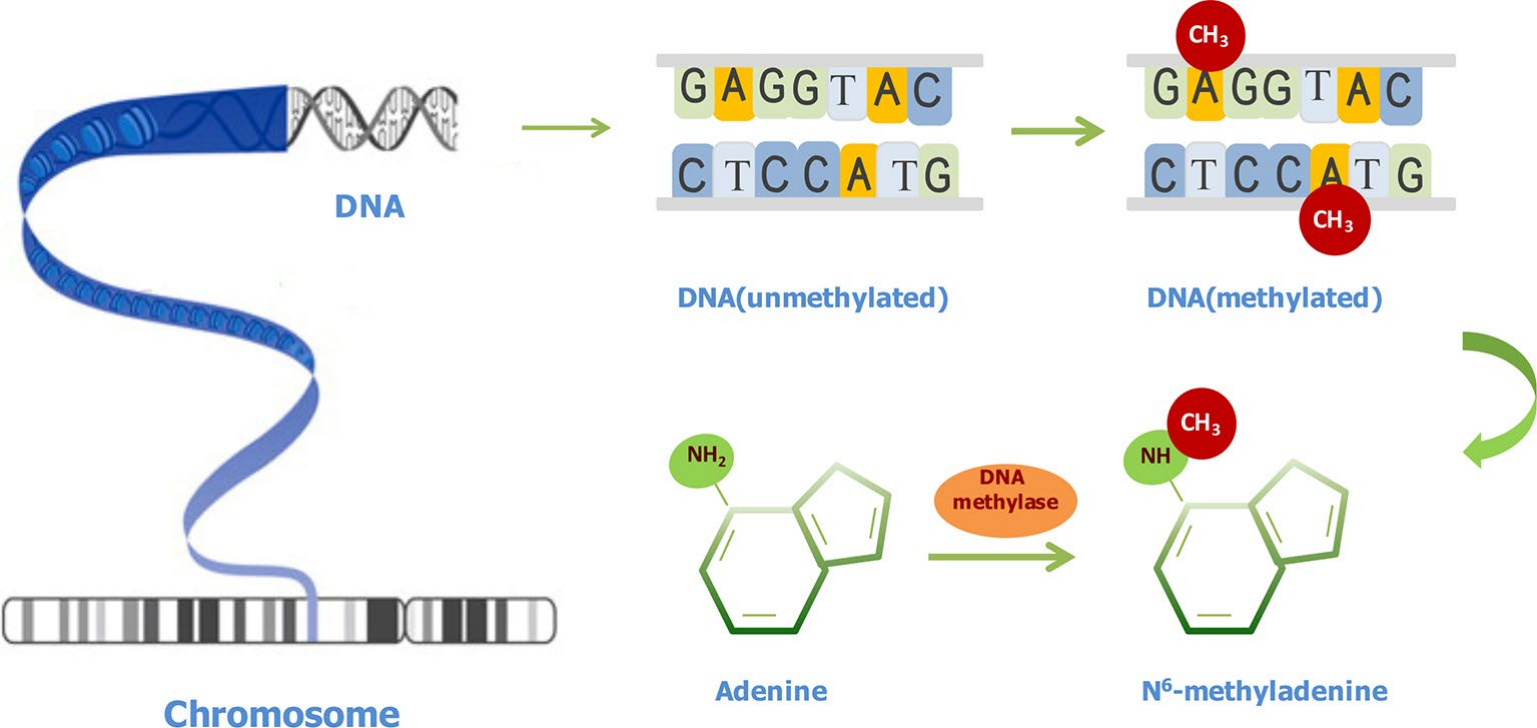
Illustration of N6-methyladenine (6mA) modifications in DNA. The conversion of adenine to 6mA is mediated by methyl-transferases.

As shown in **Figure 1**, after a methyl group is transferred to the sixth position of adenine ring, under the catalysis action of methyltransferases, 6mA is formed. 6mA is a noncanonical DNA modification form in different eukaryotes at low levels (Fu et al., 2015; Greer et al., 2015; Zhang et al., 2015; Koziol et al., 2016; Liu et al., 2016; Mondo et al., 2017; Wang et al., 2017). 6mA in prokaryotes and eukaryotes shows similar characteristics (Heyn and Esteller, 2015). It has diverse functions, including guiding the discrimination of an original DNA strand from a newly synthesized DNA strand (Wion and Casadesus, 2006), regulating gene transcription (Cheng et al., 2016), repressing transposable elements, and reducing the stability of base pairings (Fang et al., 2012). Surprisingly, the methylation protection is an inheritable state, although it may be changed by environmental factors (Wion and Casadesus, 2006). Therefore, it is worth underscoring the importance of 6mA throughout generations.

Recent studies revealed the genome-wide distributions of 6mA in *Tetrahymena* (Wang et al., 2017), *Chlamydomonas reinhardtii* (Fu et al., 2015), *Drosophila melanogaster* (Zhang et al., 2015), *Caenorhabditis elegans* (Greer et al., 2015), vertebrates (e.g. frog and fish) (Koziol et al., 2016; Liu et al., 2016), mammals (e.g., human and *Mus. musculus*) (Wu et al., 2016; Yao et al., 2017; Xiao et al., 2018; Zou et al., 2018a), fungi (Mondo et al., 2017), and vascular plants (e.g. rice) (Zhou et al., 2018). Although these studies testified the presence of 6mA in eukaryotic genomes based on experimental means and indeed achieved encouraging results, the implication of 6mA in epigenetics is still obscure (Ratel et al., 2006). In addition, in eukaryotes, the level of 6mA was so low that it could only be detected by advanced techniques. In rice, with two antibodies, based on SMRT and IP-seq, Zhou et al. (2018) found that AGG-rich sequences were the most significantly enriched for 6mA. Thus, the computational prediction of 6mA sites may be a good choice to reduce experimental costs and guide the experimental study on plant 6mA.

In fact, several computational methods have been applied in the identification of DNA methylation sites. Based on the data of experimentally confirmed 4mC sites,Chen et al. (2017) firstly developed a predictor called iDNA4mC to identify 4mC sites, in which DNA samples were formulated with nucleotide frequency and nucleotide chemical property. Then, based on the dataset (Chen et al., 2017), He et al. (2018a) established another tool named 4mCPred, and Wei et al. (2018b) built a new predictor (4mcPred-SVM) to predict 4mC sites. Recently, a free tool called iDNA6mA-PseKNC was constructed for the computational prediction of 6mA sites (Feng et al., 2019). The tool could be used to identify 6mA sites in *Mus. musculus* genome. However, the tool could not provide valuable data contained in plant genomes due to the difference between mammal and plant genomes. Thus, it is necessary to develop a 6mA site predictor for plant genomes. Recently, a tool named i6mA-Pred was constructed to identify 6mA site in rice (Chen et al., 2019). The tool could realize the area under the receiver operating characteristic curve (auROC) of 0.886 in jackknife cross-validation. However, the database used was not large enough, and the accuracy should be further improved.

**Figure 2:**
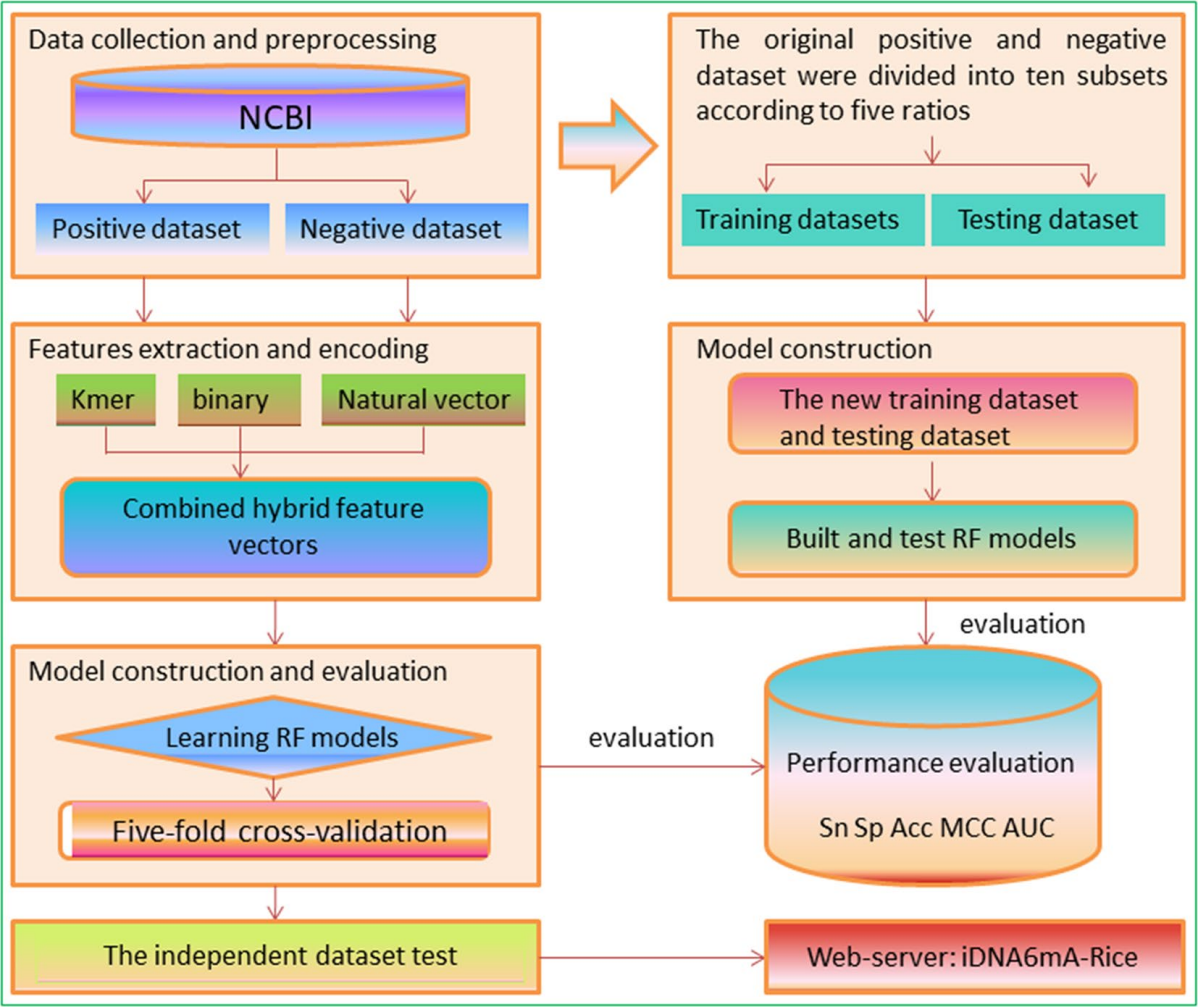
A flowchart used in this study.

In view of the aforementioned descriptions, this study aims to develop a new method and establish an efficient tool to identify 6mA sites in the rice genome. A flowchart is shown in **Figure 2**. We firstly collected the existing data in the rice genome, including experimentally confirmed non-6mA sequences and 6mA sequences and built a benchmark dataset based on the report by Zhou et al. (2018). Subsequently, three kinds of sequence encoding features were proposed to formulate samples as the input of the Random Forest algorithm (RF) to discriminate 6mA sequences from non-6mA sequences. Then, several experiments were performed to investigate the prediction capability of the proposed method. Finally, on the basis of the method, we established a predictor called iDNA6mA-Rice.

## Materials and Methods

### Benchmark Dataset

A benchmark dataset is important in building a reliable prediction model. By combining immunoprecipitation with single-molecular real-time sequencing approach, 6mA sites in the rice genome had been detected (Zhou et al., 2018) and deposited in Gene Expression Omnibus (GEO) database, which was created and is maintained by the National Center for Biotechnology Information (NCBI) (Long et al., 2019). Therefore, a total of 265,290 6mA sites containing sequences were obtained from GEO. All of these sequences in GEO are 41 nt long with the 6mA site at the center. To reduce homologous bias and avoid redundancy (Dao et al., 2018; Su et al., 2018; Tang et al., 2018a; Zou et al., 2018b; Feng et al., 2019), sequences with the similarity above 80% were excluded by using the CD-HIT program (Li and Godzik, 2006). Finally, we obtained 154,000 6mA sites-contained sequences as positive samples.

Negative samples were collected from NCBI (https://www.ncbi.nlm.nih.gov/genome/10) and according to the following three rules. Firstly, the 41-nt long sequences with adenine at the center were selected. Secondly, experimental results proved that the centered adenine was not methylated. Thirdly, Zhou et al. (2018) believed that 6mA most frequently occurred at GAGG, AGG, and AG motifs, so we statistically analyzed the ratios of GAGG, AGG, and AG motifs in positive samples and reported the result in **Table 1**. Based on the result in **Table 1**, we selected the negative samples with the same ratio of motifs so that the negative data were more objective. In this way, a large number of negative samples were obtained. In machine learning processes, imbalanced datasets lead to unreliable results. To balance positive and negative samples, 154,000 non-modified segments were randomly picked out as negative samples in model training. Finally, the benchmark dataset contained 154,000 positive samples and 154,000 negative samples. The benchmark dataset **S** is formulated as:

(1) $$S=S^{+}\cup S^{-}$$

**Table 1:** Details of the three motifs in positive samples.

Motifs	Numbers	Proportions (%)
GAGG	26,300	17.08
AGG	24,264	15.76
AG	22,206	14.42

where the **S** ^+^contains 154,000 positive samples; the **S** ^−^ contains 154,000 negative samples; ∪ is the symbol of “union” in the set theory. The benchmark dataset is available at http://lin-group.cn/server/iDNA6mA-Rice.

### Feature Descriptions

Feature extraction is a key step in establishing an excellent predictor (Song et al., 2012; Zuo et al., 2017; Stephenson et al., 2018; Manavalan et al., 2018a; Wei et al., 2018a; Manavalan et al., 2018b; Song et al., 2018b; Song et al., 2018c). The following three feature extraction techniques were adopted to formulate 6mA samples.

#### K-tuple Nucleotide Frequency Component

As a special form of PseKNC (Guo et al., 2014; Lin et al., 2014), the K-tuple nucleotide frequency component has been widely used in a variety of bioinformatics problems (Lin and Li, 2011; Yang et al., 2018b).

A DNA sequence **D** can be expressed as:

(2) $$D=R_{1}R_{2}R_{3}R_{4}\cdots R_{i}\cdots R_{L-1}R_{L},$$

where *R_i_* represents the nucleotide [Adenine (A), Thymine (T), Cytosine (C), and Guanine (G)] at the *i*th position; L is the length of sequence **D** and equals to 41 in this study. The strategy of k-tuple composition is to convert each sample into a 4*^k^* dimension vector expressed as:

(3) $$D={\left[f_{1}^{k-tuple}f_{2}^{k-tuple}\cdots f_{i}^{k-tuple}\cdots f_{4^{k}}^{k-tuple}\right]}^{T}$$

where *T* represents the transposition of the vector and $f_{i}^{k-tuple}$ represents the frequency of the *i*th *k*-tuple composition in the DNA sequence sample. The feature has been applied in DNA element identification (Wei et al., 2018b). Here, we set *k* = 2, 3, 4.

#### Mono-Nucleotide Binary Encoding

The second feature technique is to transfer nucleotide into a binary code formulated as:

(4) $$n=\{\begin{array}{ll} (1,0,0,0), & when\;n=A\\ (0,1,0,0), & when\;n=C\\ (0,0,1,0), & when\;n=G\\ (0,0,0,1), & when\;n=T \end{array}$$

Thus, an arbitrary DNA sequence with *L* nucleotides can be described as a vector of 4 × *L* features (Song et al., 2018a; Wei et al., 2018b).

#### Natural Vector

In the natural vector method proposed by Deng et al. (2011), sequences are represented as points in high-dimensional space based on statistical characteristics (Liu et al., 2018). With the sequence data, such as occurrence frequencies, the central moments, and average positions of nucleotides, the natural vector method is used to describe the distributions and numbers of nucleotides, cluster sequences, and predict their various attributes.

Based on Eq. (3), each nucleotide *R* can be defined as follows:

(5) $$W_{k}(\cdot ):\{A,C,G,T\},\to \{0,1\},$$

where *W_R_* (*R_i_*) = 1 if *D_i_* = *R* and *W_R_* (*D_i_*) = 0, otherwise

(6) $$n_{R}=\sum W_{R}(D_{i}),$$

where *n_R_* represents the number of nucleotide *R* in the DNA sequence *D*:

(7) $$S_{\left[R][i\right]}=i\cdot W_{R}(D_{i}),$$

where *S*_[_*_R_*_][_*_i_*_]_ represents the distance from the first nucleotide to the *i*th nucleotide *R*.

(8) $$T_{R}=\sum S_{\left[R][i\right]},$$

where *T_R_* represents the total distance of each set of the four nucleotides.

(9) $$\mu_{R}=T_{R}/n_{R},$$

where μ*_R_* represents the mean position of the nucleotide *R*.

Finally, the second-order normalized central moments can be defined as:

(10) $$D_{2}^{R}=\sum \frac{{\left(S_{{\left[R\right]}_{\left[i\right]}}-\mu_{R}\right)}^{2}}{nn_{R}}$$

Then, the natural vector of sequence *D* is expressed as (Tian et al., 2018):

(11) $$\left(n_{A},\mu_{A},D_{2}^{A},n_{c},\mu c,D_{2}^{C},n_{G},\mu_{G},D_{2}^{G},n_{T},\mu_{T},D_{2}^{T}\right).$$

### Random Forest Algorithm

The RF algorithm has been extensively applied in computational biology (Zhao et al., 2014; Zhang et al., 2016; Lv et al., 2019), since it is a flexible and practical machine learning method and can deal with many input variables without variable deletion and provide an internal unbiased estimate of the generalization error. According to the principle of RF, many trees are randomly generated with the recursive partitioning approach, and then, the results are aggregated according to voting rules. In this study, the number of trees is set to 100 with the seed of 1. The details of RF had been described by Breiman (2001).

### Performance Evaluation

Cross-validation test is a statistical analysis method for assessing a classifier. For the purpose of saving computation time, the fivefold cross-validation test was performed to assess the method proposed in this study. We used four metrics [Matthew’s correlation coefficient (*MCC*), sensitivity (*Sn*), overall accuracy (*Acc*), and specificity (*Sp*)] to measure the predictive capability of our model (Zuo et al., 2014; Zou et al., 2016; Manavalan and Lee, 2017; Manavalan et al., 2017; Cao et al., 2017a; Cao et al., 2017b; Cheng et al., 2018a; Yang et al., 2018a; Zhu et al., 2019).

(12) $$\{\begin{array}{cc} Sn=1-\frac{N_{-}^{+}}{N^{+}} & 0\leq Sn\leq 1\\ Sp=1-\frac{N_{+}^{-}}{N^{-}} & 0\leq Sp\leq 1\\ Acc=1-\frac{N_{-}^{+}+N_{+}^{-}}{N^{+}+N^{-}} & \;0\leq Acc\leq 1\\ MCC=\frac{1-(\frac{N_{-}^{+}}{N^{+}}+\frac{N_{+}^{-}}{N^{-}})}{\sqrt{\left(1+\frac{N_{+}^{-}-N_{-}^{+}}{N^{+}})(1+\frac{N_{-}^{+}-N_{+}^{-}}{N^{-}}\right)}} & \;0\leq MCC\leq 1 \end{array}\text{​},$$

where *N*^+^ and *N*^−^ are, respectively, the numbers of 6mA sites and non-6mA sites in benchmark dataset; $N_{-}^{+}$ indicates the number of the 6mA sites recognized as non-6mA sites; and $N_{+}^{-}$ indicates the number of the wrongly predicted non-6mA sites. *Sn* and *Sp* represent the ability of a model to correctly identify 6mA sites and non-6mA sites, respectively. The value of *Acc* indicates the overall accuracy of our model distinguishing 6mA sites from non-6mA sites. *MCC* indicates the performance of our model based on real and predicted values. When $N_{-}^{+}=N_{+}^{-}=0$, meaning that none of the 6mA sites in the dataset *S*^+^ and none of the non-6mA sites in the dataset *S*^−^ was mispredicted, we have *MCC* = 1; when $N_{-}^{+}=N^{+}/2$ and $N_{+}^{-}=N^{-}/2$, we have *MCC* = 0, meaning no better than random prediction; when $N_{-}^{+}=N^{+}$ and $N_{+}^{-}=N^{-}$ we have *MCC* = -1, meaning total disagreement between prediction and observation.

In addition to the analysis based on the previously discussed indicators, the ROC curves (Metz, 1989; Chen et al., 2016; Dao et al., 2018; Feng et al., 2018; Lai et al., 2019; Tan et al., 2019) were plotted, and then, the area under the receiver operating characteristic curve (AUC) was calculated to objectively evaluate our proposed model.

## Results and Discussion

### Sequence Analysis

**Figure 3:**
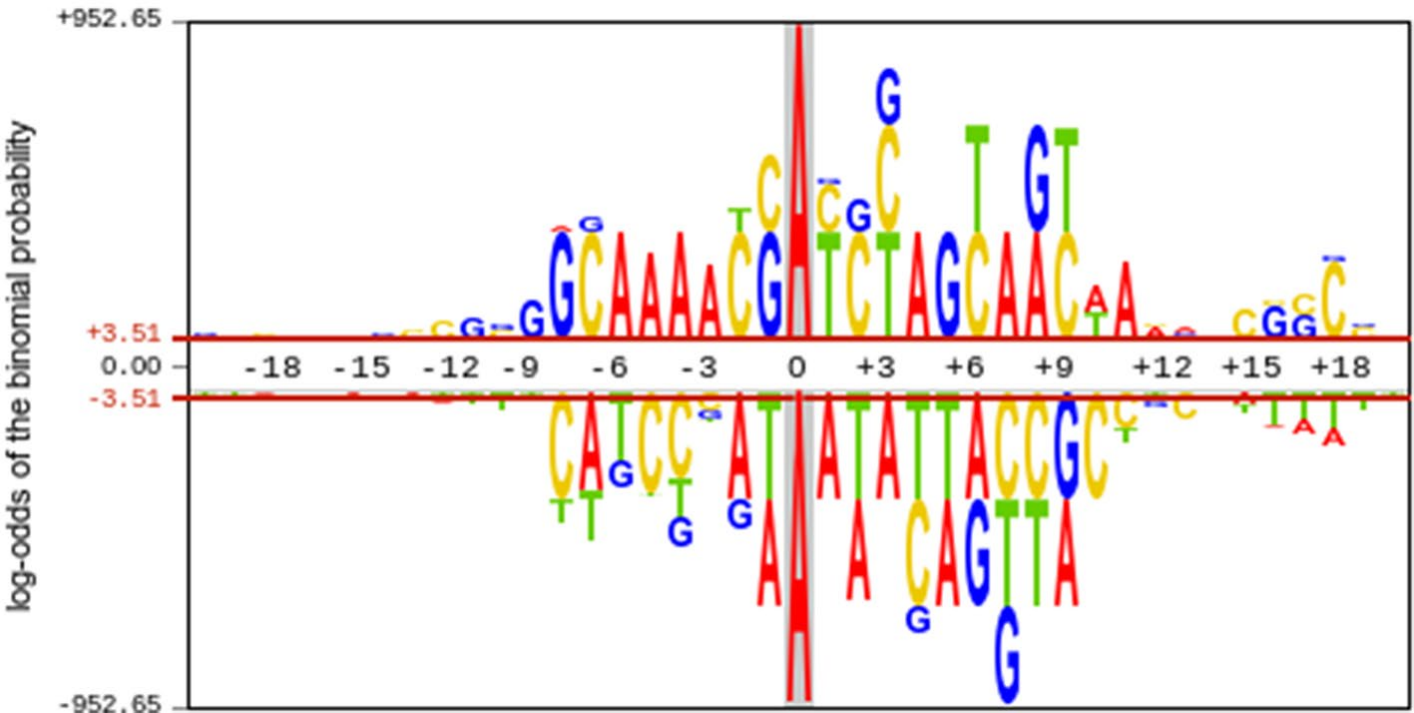
Nucleotide distribution preferences around 6mA and non-6mA sites. The upper half of the x-axis indicates the nucleotide distribution in 6mA site containing sequence, whereas the lower half of the x-axis indicates the nucleotide distribution in non-6mA site containing sequences.

To investigate the nucleotide distribution around the 21st site (6mA or non 6mA) in positive and negative samples, the pLogo (O’Shea et al., 2013) was plotted to analyze the statistical difference of nucleotide occurrence between two kinds of samples. The 6mA samples were dramatically different from non-6mA samples in terms of nucleotide compositions (**Figure 3**). The nucleotide composition bias regions existed in the ranges from -8 to +10 sites and from +15 to +18 downstream of the 6mA site. Unlike the distribution in the non-6mA samples, a consensus motif of AAAA was observed in the upstream of the 6mA site. These results suggested that it was feasible to construct a machine learning model for identifying 6mA sites with extracted sequence features.

### Performance Evaluation on Different Features

**Figure 4:**
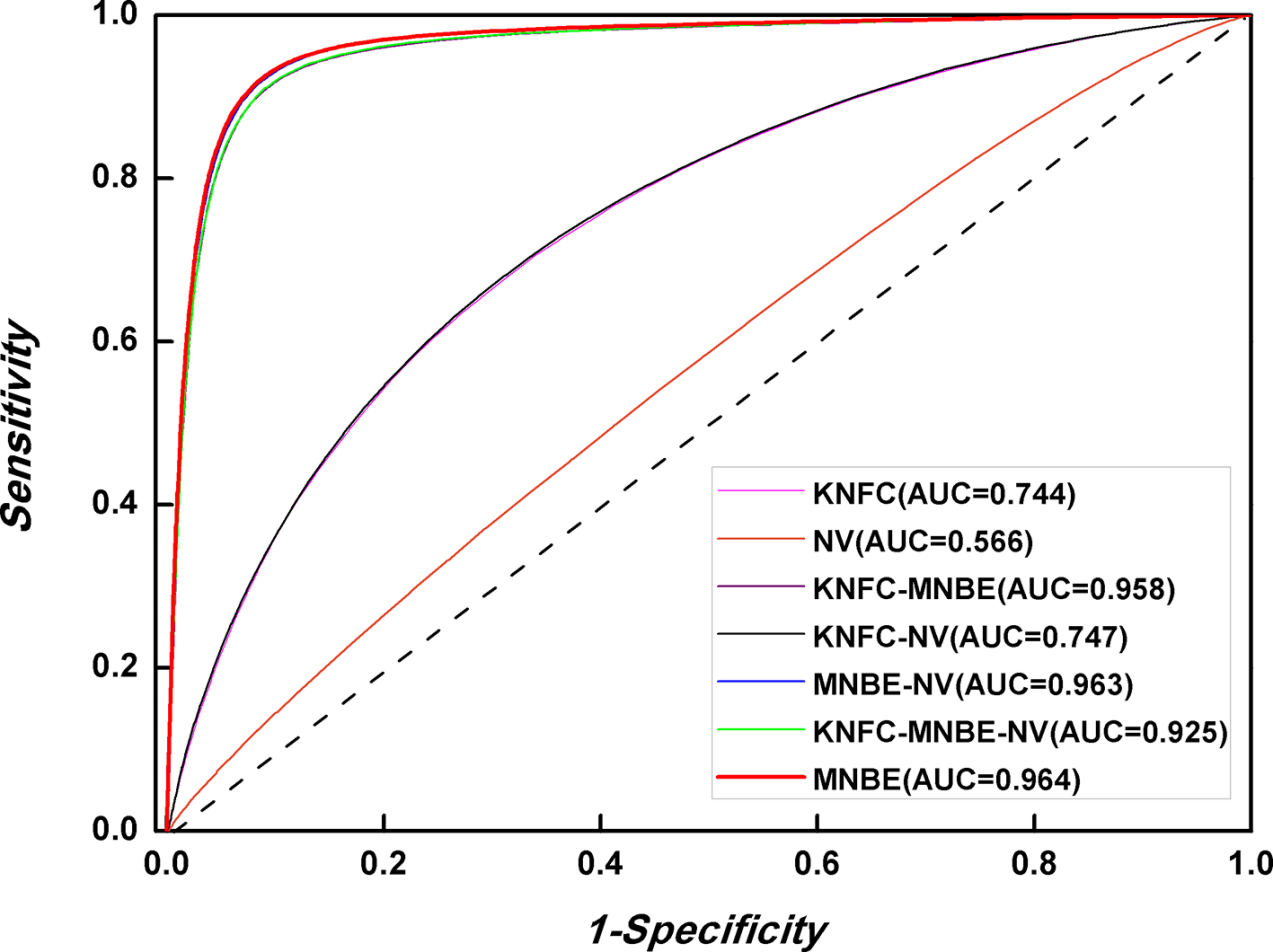
Performance evaluation based on three features and their combinations.

The prediction performances of three features [K-tuple nucleotide frequency component (KNFC), mono-nucleotide binary encoding (MNBE), and natural vector (NV)] and their combinations were firstly explored with RF. Accordingly, we built four computational models and evaluated them through the fivefold cross-validation test. The prediction results are provided in **Figure 4** and **Table 2**. It was found that MNBE could produce the best prediction performance among all features, indicating that it was the best descriptor for 6mA samples.

**Table 2:** Predictive performances of KNFC, MNBE, and NV.

Methods	*Sn* (%)	*Sp*(%)	*Acc*(%)	MCC	AUC
KNFC (k = 2, 3, 4)	70.3	66.3	68.3	0.366	0.744
MNBE	93.0	90.5	91.7	0.835	0.964
NV	58.1	50.6	54.3	0.087	0.566
KNFC-MNBE	91.8	90.1	90.9	0.819	0.958
KNFC-NV	70.4	66.5	68.4	0.369	0.747
MNBE-NV	92.8	90.3	91.6	0.832	0.963
KNFC-MNBE-NV	91.7	90.3	91.0	0.820	0.925

KNFC is a commonly used feature extractor technique and has been successfully applied in DNA regulatory element prediction. However, the results in **Table 2** showed that the accuracy of KNFC was only 68.3%, which was far from satisfactory. For the 41-nt long 6mA samples, KNFC is a high-dimension vector (16 + 64 + 256), which is so large that many elements in feature vector are zero. Although high-dimension features contain more information, more noise and redundant information are also included, thus decreasing the discrimination capability. Therefore, KNFC is not suitable for 6mA identification. In fact, the NV is the worst descriptor among all features in this study, since it can only obtain the overall accuracy of 54.3%, which almost equals the accuracy of random guess. The reason for the poor performance of NV in 6mA prediction is that NV contains too few features to capture enough sequence information of 6mA and non-6mA samples.

For the combinations of different features, if MNBE was included, the prediction performances are always good. However, they are still not higher than those obtained with MNBE alone. Thus, subsequent studies were based on MNBE.

### Performance Evaluation of Different Algorithms

**Figure 5:**
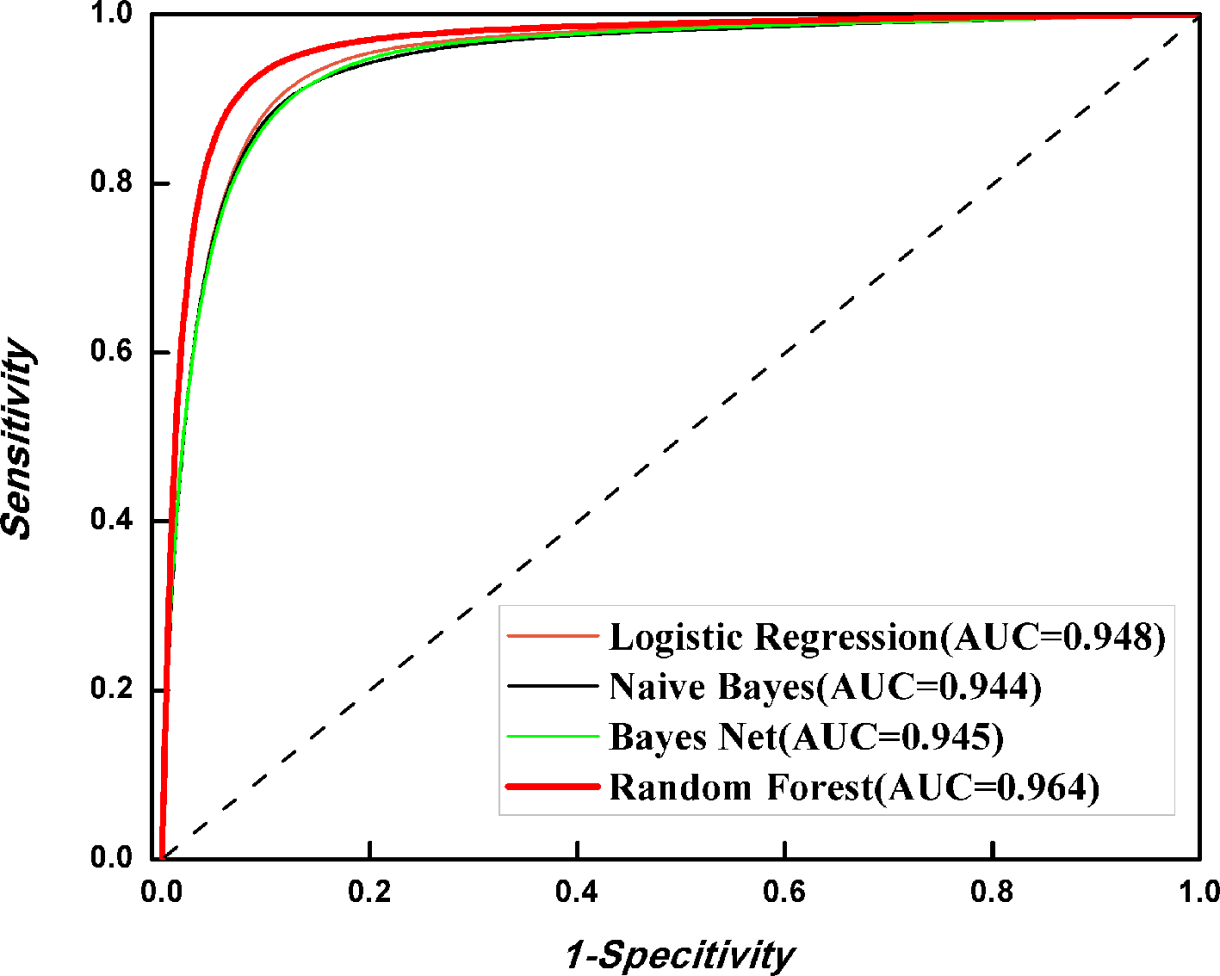
Performance evaluation of different algorithms.

It is natural to ask whether other classification is better than RF in 6mA identification. Thus, we investigated the discriminant capabilities of three algorithms, namely, Naïve Bayes, Bayes Net, and Logistic Regression, with the benchmark dataset through fivefold cross-validation. All algorithms were implemented in WEKA (Frank et al., 2004). The ROC curves were plotted (**Figure 5**). It is obvious that RF is the best one for 6mA prediction among four algorithms. Thus, the final model was built with RF.

### Performance Evaluation Based on Different Data Ratios

In order to further assess the proposed method, the benchmark dataset was randomly divided into two parts according to five ratios (5:5, 6:4, 7:3, 8:2, and 9:1): training dataset and testing dataset. The former part was used to train the model, whereas the other part was used to test corresponding model. In this way, the training dataset and testing dataset are independent of each other. The predictive results are listed in **Table 3**. For each ratio between training and testing datasets, the model could always produce the AUC of >0.90, suggesting that our method was robust and reliable.

**Table 3:** Predictive performances of five ratios on the testing and training datasets.

Ratios	5:5		6:4		7:3		8:2		9:1	
	testing	training	testing	training	testing	training	testing	training	testing	training
*Sn* (%)	91.4	91.8	92.0	91.9	92.2	92.4	92.4	92.5	92.7	92.7
*Sp* (%)	70.9	90.5	87.7	90.0	90.6	90.0	91.7	90.1	92.1	90.4
*Acc* (%)	81.1	91.1	89.9	90.9	91.4	91.2	92.1	91.3	92.2	91.8
*MCC*	0.636	0.822	0.798	0.819	0.828	0.824	0.841	0.827	0.853	0.835
***AUC***	**0.904**	**0.969**	**0.953**	**0.963**	**0.963**	**0.963**	**0.967**	**0.963**	**0.969**	**0.964**

### Performance Evaluation With an Independent Dataset

We designed the third experiment to investigate the performance of our proposed predictor. In the experiment, an independent test set was collected from NCBI Gene Expression Omnibus (https://www.ncbi.nlm.nih.gov/geo/) with the accession number GSE103145 (Zhou et al., 2018). All the sequences were 41 nt long with the 6mA site at the center. After removing redundant information with CD-HIT program according to the cutoff of 60%, a total of 880 positive samples were obtained (Chen et al., 2019). The negative samples were also obtained from the rice genome. In the report by Zhou et al., 6mA most frequently occurs at GAGG motifs and seldom occurs in coding sequences (CDSs). Thus, negative samples were extracted from CDSs with GAGG motifs in the rice genome. In total, 880 negative samples with the sequence identity less than 60% were obtained. All negative samples were also 41 nt long with non-methylated adenosine at the center. The data were utilized as the benchmark dataset in i6mA-Pred (Chen et al., 2019). The details for the benchmark dataset are available at http://lin-group.cn/server/iDNA6mA-Rice.

We utilized these data to examine our proposed model (**Table 4**). In total, 95.8% 6mA sites and 93.3% non-6mA sites were correctly identified, suggesting that the method was a powerful tool for identifying 6mA sites in rice genome.

**Table 4:** Comparison of different methods for predicting 6mA sites in independent dataset.

Method	*Sn* (%)	*Sp* (%)	*Acc* (%)	MCC	auROC
Our method	95.8	93.3	94.6	0.891	0.981
iDNA6mA-PseKNC	76.6	94.3	85.5	0.721	–

### Comparison With Published Methods

Till now, i6mA-Pred (Chen et al., 2019) is the only computational-based predictor for 6mA site prediction in the rice genome. To provide an objective and strict comparison, we investigated the performance of our method with the same data through jackknife cross-validation. The method could produce the auROC of 0.910 (**Table 5**), which was higher than that of i6mA-Pred. This comparison demonstrated that our method was powerful.

**Table 5:** Comparison of different methods for predicting 6mA sites in the rice genome with jackknife test.

Methods	*Sn* (%)	*Sp* (%)	*Acc* (%)	MCC	auROC
This study	83.86	83.41	83.63	0.67	0.910
i6mA-Pred	82.95	83.30	83.13	0.66	0.886

Subsequently, iDNA6mA-PseKNC (Feng et al., 2019) is a tool to identify 6mA sites in *Mus. musculus* genome, and it can identify 6mA sites in many other species with high success rates. Thus, it is necessary to compare our proposed method with it. We investigated the performance of our predictor and iDNA6mA-PseKNC based on the independent dataset used in this work. All compared results were recorded in **Table 4**. It is obvious that the model proposed in this paper is superior to iDNA6mA-PseKNC for identifying 6mA sites. 

### Web Server

Databases and web servers (Wang et al., 2014; Liang et al., 2017; Yi et al., 2017; Zhang et al., 2017; Cui et al., 2018; Dao et al., 2018; Cheng et al., 2018b; He et al., 2018b; Hu et al., 2019; Cheng et al., 2019a; Cheng et al., 2019b) can provide scholars with more convenient services. Thus, the basis of the novel method, we built a web server named iRNA6mA-Rice to identify 6mA sites in the rice genome. The web server can be freely accessible at http://lin-group.cn/server/iDNA6mA-Rice.

**Figure 6:**
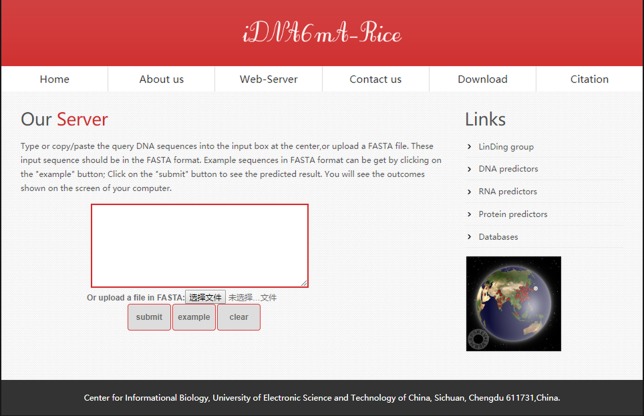
A semi-screenshot for the web server page of the iDNA6mA-Rice web server at http://lin-group.cn/server/iDNA6mA-Rice.

Users can open the homepage shown in **Figure 6** to see a short introduction about iDNA6mA-Rice. One may firstly click the “Web-server” button, then type or copy/paste DNA sequences in the input box, or upload the FASTA format file. Note that the length of each sequence should be greater than 41 nt. Subsequently, after clicking the “submit” button, the predicted results will appear on a new page. As described previously, the tool is simple and can provide a convenient way for users to identify putative 6mA sites in DNA of their interest. Moreover, in order to facilitate the processing of large-scale data, the stand-alone package can be downloaded at http://lin-group.cn/server/iDNA6mA-Rice/download.html.

## Conclusions

This paper developed a computational method for the identification of 6mA sites in the rice genome. We designed several kinds of experiments to examine the performance of the proposed method, for example, the performance evaluation on different features, performance evaluation on different algorithms, performance evaluation based on different data ratios, performance evaluation with an independent dataset, and comparison with published methods. All results demonstrated that our proposed method could accurately recognize 6mA sites in the rice genome. For the convenience of most wet-experimental scholars, we established a free web server to predict 6mA sites. We anticipate that the web server can promote the efficient discovery of novel potential 6mA sites in the rice genome and facilitate the exploration of their functional mechanisms in gene regulation.

## Data Availability

All datasets generated for this study are included in the manuscript/supplementary files.

## Author Contributions

WC, YZ, and HLin conceived the study. HLv and F-YD implemented the study and drafted the manuscript. HLv, Z-XG, and DZ wrote the custom scripts and performed analysis. HLv, WC, and YZ interpreted the data. All authors read and approved the manuscript.

## Funding

This work has been supported by the National Nature Scientific Foundation of China (grant nos. 61772119 and 31771471) and the Science Strength Promotion Programme of UESTC.

## Conflict of Interest Statement

The authors declare that the research was conducted in the absence of any commercial or financial relationships that could be construed as a potential conflict of interest.
